# Incidence and determinants of adverse outcomes among women who were managed for eclampsia in the University of Gondar Comprehensive Specialized Hospital, Northwest Ethiopia

**DOI:** 10.1186/s12884-021-04199-1

**Published:** 2021-10-29

**Authors:** Yisfa Getaneh, Elfalet Fekadu, Adamu Takele Jemere, Zelalem Mengistu, Gebrekidan Ewnetu Tarekegn, Mohammed Oumer

**Affiliations:** 1 Department of Gynecology and Obstetrics, School of Medicine, College of Medicine and Health Sciences, University of Gondar, Gondar, Ethiopia; 2 Department of Health Informatics, Institute of Public Health, College of Medicine and Health Sciences, University of Gondar, Gondar, Ethiopia; 3 Department of Epidemiology and Biostatistics, Institute of Public Health, College of Medicine and Health Sciences, University of Gondar, Gondar, Ethiopia; 4 Department of Human Anatomy, School of Medicine, College of Medicine and Health Sciences, University of Gondar, Gondar, Ethiopia

**Keywords:** Adverse maternal outcomes, Determinant factors, Eclampsia, Ethiopia

## Abstract

**Background:**

The incidence of eclampsia and its adverse maternal outcomes are very high in developing countries, particularly in Subsaharan African Countries. Identifying predictors for adverse maternal outcomes of eclampsia has paramount importance for helping health care providers to optimize their management outcomes. Therefore, this study aimed to assess the incidence of adverse maternal outcomes of eclampsia and its determinant factors.

**Methods:**

A retrospective follow-up study design was applied. The data were extracted from patient charts using a structured, pre-tested, questionnaire. Descriptive analyses (frequencies, means, and standard deviation) were calculated, and bi-variable and multivariable logistic regression models were used to testing the association between independent variables and an outcome variable. After the data were coded and entered into Epi-Info Version 7.2 Software, the data were analyzed using STATA Version 14 Statistical Software.

**Results:**

The magnitude of eclampsia was 5.36 per 1000 pregnancies (95% CI: 4.72, 6.10). The incidence of adverse maternal outcomes in eclamptic mothers was 53.7% (95% CI: 47.02, 60.24%). After adjusting for covariates maternal age 30–34, AOR 5.4 [95% CI = 1.02, 28.6]; age above 34, AOR 10.5 [95% CI = 1.3, 88.6]; gravidity 2–4, AOR 0.3 [95% CI = 0.1, 0.9]; 10 or more convulsions, AOR 4.6 [95% CI = 1.4, 14.9]; mild pyrexia, AOR 20.4 [95% CI = 3.7, 112.7]; moderate pyrexia, AOR 14.6 [95% CI = 1.7125.1]; platelet count below 50,000 cells/mm^3^, AOR 34.9 [95% CI = 3.6, 336.2]; platelet count between 50,000 and 99,000 cells/mm^3^, AOR 24.5 [95%CI = 5.4111.6]; and stillbirth of the current pregnancy, AOR 23.2 [95%CI = 2.1257.5] were strong predictors of adverse maternal outcomes in eclamptic mothers.

**Conclusions:**

The incidence of adverse maternal outcomes of eclampsia was found to be high compared to similar studies discussed in this study. This study recommends early identification of patients with the risk factors (having many convulsions, high body temperature, low platelet count, patient age above 30 years, and 2–4 pregnancies), strengthening the referral system, and advocation of research on the area of adverse maternal outcomes and thereby encourage evidence-based medicine.

**Supplementary Information:**

The online version contains supplementary material available at 10.1186/s12884-021-04199-1.

## Background

The overall global burden of eclampsia is 1.4% [1]. The incidence of eclampsia is 0.1% in Europe, 2.7% in Africa, 0.03% in the United Kingdom, 0.03 in Qatar, and 0.7% in Ethiopia [1–5]. Hypertensive disease of pregnancy (including eclampsia) is the cause for more than half of global maternal deaths together with obstetric hemorrhage and puerperal sepsis [6, 7]. Compared to developing countries (Case Fatality Rate (CFR) of India [8], Nigeria [9], and Ethiopia [5] was 17.7, 22.3, and 13.3%, respectively), the CFR of eclampsia is very low in developed countries ranging from 0% (no case fatality) up to 1.8% [3, 8, 10]. Eclampsia is also the leading factor for maternal morbidities, maternal near-miss cases are 60 times more frequent in eclamptic mothers [1, 2]. In eclamptic patients, the common adverse maternal outcomes are abruption placenta, disseminated intravascular coagulation (DIC), maternal shock, hemolysis elevated liver enzymes and low platelets syndrome (HELLP), acute kidney injury (AKI), respiratory distress, neurologic complications, postpartum hemorrhage (PPH), blood transfusion requirement, intensive care unit (ICU) admission, and maternal deaths [2, 6, 10–19].

Among different characteristics, factors significantly associated with adverse maternal outcomes of eclampsia were gestational age, platelet count, the number of convulsions, birth weight, residence, maternal age, gravidity, and the presence of stillbirths [20–24].

Essentially, morbidities and mortalities of eclamptic mothers can be prevented through the provision of early and effective medical treatments [25, 26]. Less antenatal care due to poor health system, unavailability of trained health professionals, low quality of care, low ICU, less availability of medical supplies/medications, and poverty contribute to the vulnerability of mothers to eclampsia and its complications [19, 27, 28]. As Ethiopia is one of the developing countries, morbidities and mortalities associated with eclampsia are presumed to be high. Identifying adverse maternal outcomes and their predictors could help health care providers (and policymakers) to enhance their plans and intervention strategies to prevent this serious deadly pathology and subsequent sequalae. Therefore, this study aimed to assess the incidence of adverse maternal outcomes of eclampsia and its determinant factors.

## Methods

### Study design, period, and setting

We employed a retrospective follow-up study design to assess the incidence of adverse maternal outcomes of eclampsia and its determinants at the University of Gondar Compressive Specialized Hospital (UOGCSH), Gondar, Northwest Ethiopia. UOGCSH is one of the pioneer hospitals in the country and serving the population for more than 60 years. This teaching hospital provides more than 18 undergraduate and postgraduate programs in medicine and related sciences. Besides, it serves as a referral center for more than 7 million populations with varying climatic and geographical characteristics from related provinces, regions, and countries. Department of Obstetrics and Gynecology started a specialty program in 2010. There are now 50 residents and three subspecialty programs in three fields that have just opened. Every year, roughly 200,000 individuals visit the hospital, according to the Hospital Plan and Program Information Center. The total number of deliveries each year averages 8000. In this study, the data were collected from September 01, 2013, to August 31, 2020.

### Study population and inclusion/exclusion criteria

All patients who were diagnosed with eclampsia during pregnancy or postpartum period and were admitted and managed as eclampsia at UOGCSH in the data collection period were included in the study. Patients with a previous history of epilepsy and patients with other causes for convulsions (like infection, electrolyte imbalance, toxic or metabolic encephalitis, brain tumors, and trauma) were excluded from the study.

### Sample size determination and sampling technique

The sample size was determined using the single population proportion formula by assuming a 95% level of confidence, 16% incidence [13], 5% margin of error, and adding 10% to account for incomplete/lost charts, resulting in a required sample size of 227. To get to this size, all eclamptic patients in the specified period were taken. Therefore, the final sample size included in this study was 231 patients.

The diagnosis and medical record number were searched from the morning presentation registry and all medical records/charts with eclampsia diagnosis were considered for data collection.

### Data collection procedures

A pretested and structured questionnaire was used for data collection. The data collection questionnaire was adapted from different literature [29, 30] and modified by investigators to satisfy the objectives of this study (Supplementary file 1). The clients’ demography, detailed clinical characteristics, complications, and management characteristics were extracted through chart reviewing of eclamptic patients. Data collection was done by three General Practitioners (Physicians). The medical record number of eclamptic patients in the morning registry was used to access patients’ main charts.

### Follow-up and study variables

In the beginning, after the identification of eclamptic patient charts, all baseline data were collected. Then, all patient charts were followed to observe the status of the outcome. During the follow-up, all the necessary data were recorded (clinical profile, maternal measurements, medications or procedures prescribed, clinical features, maternal complications, and diagnostic/laboratory findings, for example). At the end of the follow-up, all outcome status, clinical management information, and maternal complications were also recorded meticulously.

The adverse maternal outcomes of eclampsia were an outcome variable. It refers to maternal mortality or one or more serious complications of major organ morbidity. Eden’s criteria and WHO maternal near-miss criteria were employed for describing the severity of eclampsia. Adverse maternal outcomes are abruption placentae, DIC, maternal shock, HELLP, AKI, respiratory distress, neurologic complications, PPH, blood transfusion requirement, ICU admission, and maternal deaths.

The explanatory variables are socio-demographic, clinical, laboratory, and management. These are maternal age, residency, parity, gestational age, number of gestation, the presence of antenatal care visit, place of antenatal care, eminent symptoms, the number of convulsions, duration of convulsions, referral system to our hospital, past obstetric history, history of self-chronic illness, history of family chronic illness, vital signs at admission, blood pressure at referral, labor initiation, duration of labor, place of delivery, mode of delivery, indication for cesarean section, antihypertensive requirement, anticonvulsant requirement, and laboratory/imaging findings.

**Definitions:** Adverse maternal outcomes included maternal mortality or one or more serious complications of major organ morbidity. **Hypertensive disease of pregnancy**: Diastolic blood pressure (DBP) > 90 or systolic blood pressure (SBP) > 140 with two occasions at least 4 h apart and 1 week, after 20 weeks of pregnancy and 12 weeks of the postpartum period with ± proteinuria. **Maternal death or maternal mortality** is defined as the death of a woman while pregnant or within 42 days of termination of pregnancy, irrespective of the duration and site of the pregnancy, from any cause related to or aggravated by the pregnancy or its management but not from accidental or incidental causes. **Maternal near-miss:** The International Statistical Classification of Diseases and Related Health Problems 10th version defined a maternal near-miss case as a woman who nearly died but survived a complication that occurred during pregnancy, childbirth, or within 42 days of termination of pregnancy. **Uncontrolled seizure:** It is when there is a requirement of another anticonvulsant or reloading of Magnesium Sulfate after 15 min of an initial loading dose of Magnesium Sulfate to control recurrent seizures.

### Data processing

After data extraction was completed, the data were checked for completeness and accuracy. Then, the data were coded and entered into Epi-Info Version 7.2 Software. Finally, the data were exported into STATA Version 14 Statistical Software for analysis.

### Data analyses procedure

Univariate analyses were performed and presented as frequencies and percentages for categorical variables. Mean and Standard Deviation (SD) were reported for normally distributed data. The median and interquartile ranges were considered for non-normally distributed data. Bi-variable and multivariable logistic regression models were used to testing the association between independent variables and an outcome variable. Bivariate analyses were made between independent variables and the outcome variable (adverse maternal outcomes of eclampsia) and the variables with a *P*-value < 0.2 were selected for multivariable analysis. A P-value < 0.05 (95% confidence interval) was taken as a cut-off point for a statistically significant variable in multivariable logistic regression analysis. The goodness of fit was assessed by using the Hosmer and Lemeshow test.

### Data quality control

A standardized data collection tool was employed, which is adopted and modified contextually. The data collection instrument was pretested for clarity and appropriateness before the actual data collection. To ensure the quality of data, the collected data were checked daily for completeness, accuracy, and clarity by the investigators. Data clean-up and crosschecking were done before analyses. The training was given to the data collectors for 3 days to equip them with the necessary skills. The investigators continuously supervised during the data collection process and the collected data were entered on the same day to address inconsistencies regarding wrong entries.

### Ethical considerations

Ethical clearance/approval was obtained from the ethics committee of the School of Medicine, University of Gondar. With this clearance, formal approval was sought and the permission for conducting the study (official letter) was secured from the administration of UOGCSH and the Department of Gynecology and Obstetrics before commencing the study. “Informed Consent waiver was obtained from the ethics committee of School of Medicine, University of Gondar” (Dr. Abebe Muche, Chair of Ethical Review Board, reference number: 2169/08/2020) and hospital directors (Signed and approved on reference number, 2169/08/2020). The confidentially of the data gathered was kept and handled during all phases of research activities. We confirm that all methods were performed following the relevant guidelines and regulations (institutional, national, and international).

## Results

### Baseline characteristics of the participants

**Fig. 1:**
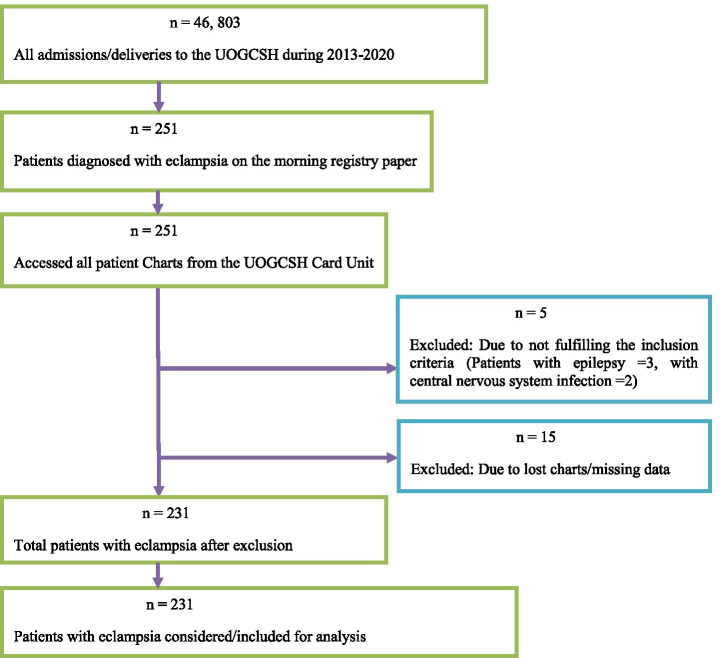
Flow diagram of patient selection at UOGCSH, 2020

The total deliveries in the study period were 46, 803, of which 251 patients were diagnosed with eclampsia and making the magnitude of eclampsia 5.36 per 1000 pregnancies (95% CI: 4.72, 6.10). Of 251 eclamptic patients, 20 were excluded due to missing data/lost charts or not fulfilling the inclusion criteria, leaving 231 patients for analyses (Fig. 1). The mean age was 24.77 years (range = 15–45 years and SD = 6.06). The majority, 206 (89.2%), were from rural areas with a range of 20–24 years. Teenagers accounted for 17.3% and those aged above 34 years accounted for 11.3% (Table 1).

**Table 1:** Baseline, clinical, and obstetric characteristics of the study participants, 2020

Characteristics	Frequency	Percent
Age (in years)		
< 20	40	17.3
20–24	82	35.5
25–29	56	24.2
30–34	27	11.7
> 34	26	11.3
Residency		
Urban	25	10.8
Rural	206	89.2
Previous obstetrics complications		
No	57	24.7
Yes	36	15.6
Others^a^	138	59.7
Chronic illness – Personal		
No	229	99.1
Yes	2	0.9
Chronic illness – Family		
No	227	98.3
Yes	1	0.4
Others^a^	3	1.3
Gravidity		
Primigravida	138	59.7
Multigravida	93	40.3
Gestational age		
Abortion	8	3.5
Preterm eclampsia	27	11.7
Term eclampsia	26	11.3
Others^a^	170	73.6
Gestations		
Singleton pregnancy	213	92.2
Twin pregnancy	18	7.8
Antenatal care visit		
Has no ANC visit	53	22.9
Has at least one ANC visit	178	77.1
Place of antenatal care		
UOGCS hospital	3	1.3
Health centers	147	63.6
Primary hospitals	17	7.4
Others (unknown)	64	27.7
Eminent symptoms		
No eminent symptoms	44	19.0
Has eminent symptoms	187	81.0
Convulsion duration		
< 3 h	15	6.5
3–5 h	55	23.8
6–11 h	83	35.9
> 11 h	78	33.8
Number of convulsions		
≤ 10 convulsions	198	85.7
> 10 convulsions	33	14.3
Time of convulsion		
Antepartum	147	63.6
Intra-partum	31	13.4
Postpartum	53	22.9
Referral system		
Come directly or no referral	31	13.4
Arrived after one health facility visit	177	76.6
Arrived after two health facility visit	23	10.0
SBP at primary care (*n* = 178)		
SBP ≤ 139 mmHg	43	24.2
SBP 140–159 mmHg	62	34.8
SBP 160–199 mmHg	69	38.8
SBP ≥ 200 mmHg	4	2.2
SBP at admission		
SBP < 140	61	26.4
SBP 140–159 mmHg	109	47.2
SBP 160–199 mmHg	61	26.4
Labor initiation		
Spontaneous	104	45.0
Induced	105	45.5
Had not to labor at all	22	9.5
Total labor duration		
No labor	22	9.5
Labor from 1 to 8 h	93	40.3
Labor from 9 to 16 h	79	34.2
Labor from 16 to 24 h	30	13.0
Labor more than 24 h	7	3.0
Mode of delivery		
Vaginal delivery	132	57.1
Cesarean delivery	99	42.9
Cesarean section indication (*n* = 99)		
Fetal tachycardia	9	9.1
Fetal bradycardia	16	16.2
Other CTG pathology	17	17.2
Failed induction	23	23.2
Cephalo pelvic disproportion	7	7.1
Primary	21	21.2
Other^a^	6	6.1
Anticonvulsants is given		
No	2	0.9
Yes	229	99.1
Reasons for use of other anticonvulsants/why not only Magnesium Sulfate (*n* = 30)		
Uncontrolled seizure	11	36.7
Lack of Magnesium Sulfate	2	6.7
Contraindicated eclampsia complications for giving Magnesium Sulfate	17	56.6

Key:^a^ = missing values or status is not known/unknown

### Clinical and obstetric characteristics

About 59.7% of patients were primigravidas and 92.2% of patients had a singleton pregnancy. Around three-fourth (77.1%) of them had at least one antenatal care visit and the majority (147 patients) had antenatal care follow-up at a nearby health center (Table 1).

Of all, 81% had prior premonitory symptoms before a convulsion, of which the headache was the most common symptom (76%), followed by the blurring of vision (38%) and vomiting (15%). Regarding the time of convulsion, 63.6, 13.4, and 22.9% were occurred in antepartum, intrapartum, and postpartum, respectively. Of all, about 34% arrived after 11 h of convulsion, 14.3% arrived after convulsion of more than 10 episodes, 83.5% experienced the first convulsion at home, and 11.7% experienced convulsion during transportation. In the present study, 86.6% of patients arrived after they were referred from one or two health facilities. Among the referrals (200 patients), 72% (144 patients) got anticonvulsants and the remaining 28% (56 patients) did not get anticonvulsants before referral. Among patients who were given anticonvulsants (144 patients), Magnesium Sulfate was given for 132 patients (91.7%) in which at least the loading or the loading and maintenance dose was administered and diazepam drip was given for 8.3% (12/144) of patients. The most common reason for referring eclampsia cases was due to inadequate setup and human power for managing eclampsia (76%) followed by the absence of basic workups for preeclampsia-eclampsia syndromes (47%). Of all eclamptic patients, 45% had spontaneous labor initiation, 3% had labor duration above 24 h, 12.5% (29 patients) had a history of home delivery, and 42.9% had a cesarean delivery. Of 99 cesarean deliveries, 78.8% (78 patients) were done at the intrapartum after labor initiation. The most common indication for cesarean delivery was failed induction (23.2%). Among the other six cases, cesarean sections were performed for cases of thick meconium in early labor, cord prolapse, antepartum hemorrhage with active bleeding and hemodynamic instability, and uterine rupture after induction with oxytocin. From the referred patients (200 patients), the blood pressure of 178 was measured. Among cases that had SBP of 160–199 mmHg (69 patients), 36 patients did not get antihypertensive at primary care. Moreover, of four patients with SBP above 200 mmHg, one patient did not get an antihypertensive drug at primary care. At admission, of 231 patients, 26.4% had SBP < 140 mmHg and 47.2% had SBP between140 and 159 mmHg. After admission, 23% (14/61) of patients with SBP < 140 mmHg and 62.4% (68/109) of patients with SBP with 140–159 mmHg required anti-hypertensive for elevated blood pressure in the severe range. Of anti-hypertensive medication (143 required), the most common anti-hypertensive was Hydralazine (prescribed for 93% of patients) followed by Methyldopa (prescribed for 60.1%) and Nifedipine (prescribed for 25.2%). Of all patients, 229 took anticonvulsant drugs at admission, and Magnesium Sulfate was the first drug of choice. Furthermore, Diazepam, Phenobarbital, Phenytoin, Mannitol, and other anticonvulsants were either added or given as alternatives to Magnesium Sulfate in 30 patients (Table 1).

With respect to perinatal outcomes, there were 18 sets of twin pregnancies, 194 live births, 42 early neonatal deaths, 18 abortions, and 27 stillbirths.

### Laboratory characteristics

In this study, two patients had severe anemia, and 20 patients had thrombocytopenia < 50,000 (Table 2).

**Table 2:** Laboratory related characteristics, 2020

Characteristics	Frequency	Percent
Hematocrit		
No anemia (≥ 33.0%)	192	83.1
Mild anemia (30.0–32.9%)	24	10.4
Moderate anemia (21.0–29.9%)	13	5.6
Severe anemia (< 21.0%)	2	.9
Platelet count		
Platelet count < 50,000 cells/mm^3^	20	8.7
Platelet count 50,000–99,000 cells/mm^3^	31	13.4
Platelet count 100,000–149,000 cells/mm^3^	44	19.0
Platelet count > 150,000 cells/mm^3^	136	58.9
SGOT		
≤ 70 u/L	139	60.2
71–175 u/L	48	20.8
176–350 u/L	12	5.2
351–525 u/L	12	5.2
≥ 526 u/L	9	3.9
Other^a^	11	4.8
SGPT		
≤ 70 u/L	168	72.7
71–175 u/L	25	10.8
176–350 u/L	17	7.4
351–525 u/L	3	1.3
≥ 526 u/L	6	2.6
Other^a^	12	5.2
BIL-T		
≤ 1 mg/dl	10	4.3
1.10–5.99 mg/dl	1	.4
≥ 6.00 mg/dl	5	2.2
Other^a^	215	93.1
LDH		
≤ 600 u/L	2	.9
≥ 601 u/L	10	4.3
Other^a^	219	94.8
Creatinine		
≤ 1.5 mg/dl	200	86.6
1.6–2.0 mg/dl	5	2.2
2.1–2.9 mg/dl	6	2.6
≥ 3.00 mg/dl	8	3.5
Other^a^	12	5.2

Key: *SGOT* Serum Glutamic Oxaloacetic Transaminase, *SGPT* Serum Glutamic Pyruvic Transaminase, *BIL-T* Total Bilirubin, *LDH* Lactic Acid Dehydrogenase; ^a^ = missing values or status is not known

### Adverse maternal outcome characteristics

Half of the patients [53.7% (124): 95% CI: 47.02, 60.24%] developed serious maternal complications (near-miss or maternal death) (Table 3). Twenty-four patients required admissions to ICU, and there were 18 maternal deaths, making the CFR of 7.8% in the study period (Table 3). Among improved patients (184), the majority of patients (116) stayed in the hospital for 4–7 days.

**Table 3:** Adverse maternal outcome characteristics of eclampsia, 2020

Characteristics	Frequency	Percent
Blood transfused		
No	214	92.6
Yes	17	7.4
Abruption placenta		
No	206	89.2
Yes	25	10.8
DIC		
No	228	98.7
Yes	3	1.3
Maternal shock		
No	219	94.8
Yes	12	5.2
AKI		
No	206	89.2
Yes	25	10.8
HELLP		
No	193	83.5
Yes	38	16.5
PPH		
No	212	91.8
Yes	19	8.2
Respiratory distress		
No	188	81.4
Yes	43	18.6
Neurologic complications		
No	166	71.9
Yes	65	28.1
ICU admission		
No	207	89.6
Yes	24	10.4
Maternal death		
No	214	92.6
Yes	18	7.8

### Determinants of adverse maternal outcomes

In this study, at the start of the analyses, the variables such as maternal age, residency, gravidity, antenatal care follow-up, place of antenatal care, number of gestation, eminent symptoms, the number of convulsions before admission, duration of convulsion before admission, the time of convulsion, the place of convulsion, the number of referrals, medications are given at primary care, previous bad obstetric outcomes, mental status at admission, blood pressure at admission, body temperature, labor initiation, labor duration, mode of delivery, and antihypertensive requirement after admission were entered separately into binary logistic regression analyses to test the association between these variables and outcome variable. Moreover, from laboratory investigations, hematocrit value, platelet count, SGOT, SGPT, Bil-T, creatinine, urine protein, and urine ketone were selected for bivariate analyses. We did not consider the variables for analysis that had missing values.

Consequently, maternal age, gravidity, the number of convulsions, rise in body temperature, low platelet count, stillbirths of current pregnancy, mode of delivery, and labor duration were significant predictors of adverse maternal outcomes of eclampsia in the outputs of binary logistic regression analyses (at *P*-value of < 0.2).

To rule out the confounding factors, covariates that display significant association in binary logistic regression analyses were entered into a multivariable logistic regression analysis. Six variables were found to be significantly and independently associated and these are maternal age (30–34 years and above 34 years), gravidity 2–4, the number of convulsions, maternal body temperature (mild pyrexia and moderate pyrexia), platelet count (< 50,000 and 50,000-99,000), and stillbirth of the current pregnancy (Table 4).

**Table 4:** Results of regression analysis showing the association between covariates and adverse maternal outcomes

Variables	Adverse maternal outcomes		COR (95% CI)	AOR (95% CI)
	No (%)	Yes (%)		
Maternal age				
< 20	20(8.66)	20(8.66)	1.0	1.0
20–24	40(17.32)	42(18.18)	1.05 (0.49, 2.24)	0.94 (0.34, 2.59)
25–29	33(14.29)	23(9.96)	0.70 (0.31, 1.58)	0.66 (0.20, 2.20)
30–34	9(3.90)	18(7.79)	2.0 (0.73, 5.50)^§^	5.39 (1.02, 28.63)*
> 34	5(2.16)	21(9.09)	4.20 (1.32, 13.34)^§^	10.52 (1.25, 88.57)*
Gravidity				
Primigravida	68(29.44)	70(30.30)	1.0	1.0
2–4	32(13.85)	28(12.12)	0.85(0.46, 1.56)	0.29 (0.09, 0.88)*
> 4	7(3.03)	26(11.26)	3.61(1.47, 8.86)^§^	0.82 (0.15, 4.37)
Number of convulsions				
≤ 10	101(43.72)	97(41.99)	1.0	1.0
> 10	6(2.60)	27(11.69)	4.69 (1.85, 11.85)^§^	4.62 (1.44, 14.88)*
Temperature				
Normal (≤ 37.5 °C)	104(45.02)	83(35.93)	1.0	1.0
Mild pyrexia (37.6–38.2 °C)	2(0.87)	22(9.52)	13.78(3.15, 60.31)^§^	20.44 (3.71, 112.7)*
Moderate pyrexia (38.3–39.9 °C)	1(0.43)	18(7.79)	22.55(2.95, 172.46)^§^	14.63 (1.71, 125.1)*
Severe pyrexia (≥ 40 °C)	0(0.0)	1(0.43)	1(−)	1(−)
Platelet count (cells/mm^3^)				
< 50,000	1(0.43)	19(8.23)	24.80 (3.23, 190.56)^§^	34.86 (3.61, 336.2)*
50,000-99,000	3(1.30)	28(12.12)	12.18 (3.53, 42.01)^§^	24.46 (5.36, 111.6)*
100,000-149,000	26(11.26)	18(7.79)	0.90 (0.45, 1.80)	0.70 (0.28, 1.77)
≥ 150,000	77(33.33)	59(25.54)	1.0	1.0
Stillbirth				
No	106(45.89)	98 (42.42)	1.0	1.0
Yes	1(0.43)	26 (11.26)	28.12 (3.75, 211.2)^§^	23.18 (2.09, 257.5)*
Mode of delivery				
Vaginal delivery	54 (23.38)	78 (33.77)	1.0	1.0
Cesarean delivery	53 (22.94)	46 (19.91)	0.60 (0.36, 1.02)^§^	1.16 (0.50, 2.71)
Total labor duration				
No labor	14 (6.06)	8 (3.46)	1.0	1.0
Labor from 1 to 8 h	50 (21.65)	43(18.61)	1.51 (0.58, 3.93)	1.13(0.29, 4.41)
Labor from 9 to 16 h	30 (12.99)	49 (21.21)	2.86 (1.07, 7.62)^§^	1.88(0.44, 8.14)
Labor from 16 to 24 h	11 (4.76)	19 (8.23)	3.02 (0.96, 9.48)^§^	1.86(0.36, 9.58)
Labor > 24 h	2 (0.87)	5 (2.16)	4.38 (0.68, 27.98)^§^	0.46(0.03, 7.13)

Key: ^**§**^ *P*-value < 0.2, *P-value < 0.05, *COR* Crude Odds Ratio, *AOR* Adjusted Odds Ratio, *CI* Confidence Interval

## Discussion

This hospital-based retrospective follow-up study was performed to identify the incidence and determinants of adverse maternal outcomes among eclamptic patients. Accordingly, a high incidence of adverse maternal outcomes was found and maternal age, gravidity, multiple episodes of convulsions, rise in body temperature, low platelet count, and stillbirth of the current pregnancy were found to be determinant factors for the development of adverse maternal outcomes.

In this study, among 46, 803 deliveries in the 8 years, the magnitude of eclampsia was 0.54%. This finding is comparable with the study done at Yekatit 12 Hospital 0.31% [31], Ethiopian referral hospitals 0.71% [24], Addis Ababa 0.72% [6], Tikur Anbessa Hospital 0.57% [32], and New Delhi 0.45% [33]. However, the finding is higher than the finding of the studies conducted in developed countries and lower than African regional estimates [1, 3, 12, 15].

In our study, adverse maternal outcomes occurred in 53.7% (95% CI: 47.02, 60.24%) of eclamptic mothers. This finding is in line with the study done in Ethiopia, 53% [34]. Nevertheless, our finding is higher than the study done in India, 16.9% [33], Kuwait, 27% [17], and WHO, 14.4% [2]. This disparity may be due to the differences in sample size, time, setup, study population, good antenatal and obstetric care, the quality and standard of care available, and the presence of modern well-equipped maternity hospitals.

Maternal age, gravidity, multiple episodes of convulsions, rise in body temperature, low platelet count, and stillbirth of the current pregnancy were the factors associated with adverse maternal outcomes. In this study, the odds of developing complications after the onset of eclampsia were ten times more common in the age group above 34 years compared to ages below 20 years, AOR 10.5 [95% CI = 1.3, 88.6]. The age group 30–34 years had a five times risk of developing adverse maternal outcomes as compared to their counterparts, AOR 5.4 [95% CI = 1.02, 28.6]. However, for the age group 20–29 years, there was no significant difference in developing adverse maternal outcomes as compared to less than 20 years. Similar studies that were done in Pakistan, China, and Uganda are in line with our study findings [35–37]. This may be due to an increased risk of associated cardiovascular and neurologic changes with age. Therefore, it has paramount importance to educate all women (particularly women of advanced age) to have antenatal care and stressing birth preparedness and emergency readiness during their antenatal care. Increasing early detection and antenatal screening will reduce the disorder and associated morbidity and mortality of the mothers. Patients with such age should deliver at a well-equipped health care facility and hospital management of labor is essential.

In this study, gravidity was significantly correlated with adverse maternal outcomes of eclampsia. Women with 2–4 pregnancies are 71% less likely to be affected by adverse maternal outcomes compared to primigravidas, AOR 0.29 [95% CI = 0.1, 0.9]. Nevertheless, there was no significant association between adverse maternal outcomes of eclampsia and the number of pregnancies above four. This finding is similar to the study conducted in Nigeria, Pakistan, Eastern Ethiopia, Egypt, Uganda, and Addis Ababa [10, 34, 35, 37–39]. One of the pathophysiologic mechanisms of preeclampsia-eclampsia is immunological maladaptive tolerance or loss of tolerance between maternal, paternal (placental), and fetal tissues. If the first pregnancy is complicated by the hypertensive disorders of pregnancy, the risk of recurrence will increase, will occur at early gestation, and will become more severe form in the next pregnancy. However, if the first pregnancy is not complicated especially by eclampsia, the incidence of eclampsia will be low and as a result the complications of it too. So that it could be the reason why multiparity is protective from developing severe/complicated forms of eclampsia even if gravidity above four is not protective. It could be explained by the fact that when mothers have many numbers of pregnancies and deliveries, the patient’s age will gradually increase too. Therefore, for gravidas above four pregnancies, associated increasing age could be the cause for not showing significant difference/not protective in developing severe morbidities when compared to primigravidas. There also could be another explanation for this. Besides, the mechanism of how patient sequalae develop may not be explained by similar pathogenesis for these different gravidity groups. So we recommend other further scientific studies regarding this specific aspect.

In our study, there was a delay in reaching tertiary level medical care after the onset of convulsions, about 14.3% of patients came to tertiary care after having more than ten convulsions. The number of convulsions before admission was significantly correlated with the development of adverse maternal outcomes. Thus, patients with more than ten convulsions had a five times greater risk of developing adverse maternal outcomes than patients with less than ten convulsions, AOR 4.6 [95% CI = 1.4, 14.9]. Similarly, in a study done in Nepal about adverse maternal outcomes and their associated factors in 2018, the number of convulsions played a significant role in the prognosis of the eclamptic mother [40]. This strong association could be explained by the fact that as patients frequently convulse, the risk of hypoxia, aspiration, and trauma during the episode of convulsion could predispose the patient to more morbidities and mortality. Furthermore, indirectly, repetitive convulsions along stay at home or other care center areas without appropriate intervention may predispose them to severe sequelae. Delays in maternal transport/late admissions may be due to inadequate roads for ambulance transportation and inadequate availability of ambulance services for remote areas. Thus, a strong collaboration of stakeholders is needed to improve the transportation and referral system. Goal-oriented training (capacity building) for health professionals to strengthening health care facilities (and improving access to maternal health facilities) for early detection and management of maternal adverse outcomes related to eclampsia. Twenty-eight percent of eclamptic patients who were referred did not get anticonvulsant. This may be due to the unavailability of anticonvulsants at the primary health care facility. However, the WHO recommends the availability of anticonvulsants should be greater than 90%, primarily Magnesium Sulfate, at any level of care [19]. In this study, Magnesium Sulfate availability/utilization was better than (could be due to a time change with facility improvement) a study done in 2011 in our country which was only 54% but still less than from WHO recommendation [19]. Magnesium Sulfate is safer and more effective when compared to other anticonvulsants for the prevention of recurrent convulsions and adverse maternal outcomes of eclampsia [25, 26]. More than 50 % of patients who were referred with SBP greater than 160 mmHg also did not get any form of antihypertensive due to lack of the drugs. The availability of antihypertensive medication in a previous Ethiopian study was 78% which is higher than our findings [19]. So adequate provision of drugs, as well as on-time management and referral of patients, is recommended at the primary health care facility.

Another finding from the clinical characteristics was the patient’s body temperature; it had a strong positive association with adverse maternal outcomes of eclampsia. In patients with mild-grade fever, the odd of developing adverse maternal outcomes were 20 times higher as compared to the patients with normal body temperature, AOR 20.4 [95% CI = 3.7, 112.7]. The odds of developing adverse maternal outcomes in patients with high-grade fever were 15 times more likely to occur as compared to their counterparts, AOR 14.6 [95% CI = 1.7, 125.1]. As Fugate et al. explained, this may be because the raised temperature indicates a significant dysfunction of the auto-regulatory capability of the brain. Meaning, the brain of eclamptic mothers may become unable to regulate the body temperature and indirectly could show severe neurologic sequelae is happening [41, 42]. Alternatively, there could be systemic infections that predispose the mother to the development of eclampsia during pregnancy [43]. Or, it could be the consequence of sepsis in organ systems such as the lung and the uterus leading to septic shock as most of the patients come late in our setup, as only 6.5% arrived at our setup within 3 h of convulsion and 30% came after 12 h of convulsion.

Platelet count level had also a pivotal role in determining adverse maternal outcomes after the onset of eclampsia. The odds of developing adverse maternal outcomes were 34 times higher in patients with platelet count less than 50, 000 cells/mm^3^, AOR 34.9 [95% CI = 3.6, 336.2]. Besides, the odds of developing adverse maternal outcomes in patients with a platelet count of 50,000–99,000 cells/mm^3^ were 24 times higher as compared to their counterparts, AOR 24.5 [95% CI = 5.4, 111.6]. This study finding is strongly in line with other studies done in Zimbabwe (adverse maternal outcomes were increased by 46 times and 19 times for platelet value less than 50,000cells/mm^3^ and 50,000–99,000cells/mm^3^, respectively) and Morocco (adverse maternal outcomes were increased by 13 times for platelet values < 50,000cells/mm^3^ compared to women with normal platelet counts) [21, 27]. This strong association could be because a very low platelet count may lead to an increased risk of bleeding in the brain (stroke, intracranial hemorrhage, associated raised intracranial pressure, brain herniation, and other severe neurologic complications) and in other organs. Eventually, these risks may predispose to maternal morbidity and mortality.

From the perinatal outcomes, mothers with stillbirth fetuses had a 23 times higher risk of developing maternal complications compared to other neonatal outcomes, AOR 23.2 [95% CI = 2.1, 257.5]. This result is similar to the study done in Pakistan and Addis Ababa, Ethiopia [32, 37]. Both studies showed a significant association between maternal death and negative fetal heartbeat status. This strong association could be because thromboplastins and other inflammatory cytokines that are released by the blood clots, damaged placenta, and dead fetus due to intrauterine fetal death/stillbirth exacerbate neuronal excitation and other maternal complications and further predispose patients for eclampsia associated adverse sequelae [44]. The occurrence of sepsis due to intrauterine fetal death/stillbirth may further deteriorate maternal morbidity and mortality [44]. In our setup, almost all cases with stillbirth came late and 90 % of these cases with stillbirth came after more than 6 h of the first convulsions.

As a strength and limitation of the study: this study is a pioneer in conducting a retrospective chart review of 8 years at this center on the specified problem in this specified long time. Although we have done the study vigorously and meticulously to improve the accuracy of the data, because of its retrospective nature, some data or variables were difficult to get and were not analyzed at all. This is because they were not documented or they were missing data sets on the chart. Due to the limited resources, some measurements/laboratory investigations may not be available for all women. However, this had no greater impact on diagnosis and outcome since other clinical-based criteria were applied, and the findings found are crucial to provide evidence to plan and implement policies and programs to improve maternal morbidities and mortalities.

## Conclusions and recommendations

Adverse maternal outcomes in eclamptic patients were found to be high. Determinants such as multiple episodes of convulsions, increased maternal body temperature, a decrease in platelet count, the presence of coexisting stillbirth, patient age group above 30 years, and having 2–4 number of pregnancies were independently associated with adverse maternal outcomes.

It is important for the government to maintain a quick referral system to avoid delays, and the quality of antenatal care should be improved at all levels to discover high-risk patients early. Furthermore, health workers should be trained in the early detection and management of eclampsia-related adverse outcomes. To health care providers, identified high-risk groups (or identified risk factors) need special attention during pregnancy and delivery. Future studies using a multi-center and prospective design, to academicians/researchers, could enable a more comprehensive list of potential independent factors to be studied in order to improve evidence-based medicine and reduce poor care quality and outcomes.

## Supplementary Information

**Additional file 1.** English version questionnaire.

## Data Availability

The data sets used and/or analyzed during the current study are available from the corresponding author on reasonable request.

## Abbreviations

AKI: Acute Kidney Injury
AOR: Adjusted Odds Ratio
CI: Confidence Interval
COR: Crude Odds Ratio
CFR: Case Fatality Rate
DIC: Disseminated Intravascular Coagulation
DBP: Diastolic Blood Pressure
MD: Medical Doctor
UOGCSH: University of Gondar Comprehensive Specialized Hospital
HELLP: Hemolysis Elevated Liver Enzymes and Low Platelets Syndrome
ICU: Intensive Care Unit
PPH: Postpartum Hemorrhage
SD: Standard Deviation
SBP: Systolic Blood Pressure
WHO: World Health Organization
