# Supplementary material for: Incidence and determinants of adverse outcomes among women who were managed for eclampsia in the University of Gondar Comprehensive Specialized Hospital, Northwest Ethiopia
Source: BMC Pregnancy Childbirth. 2021 Oct 29;21:734. doi: 10.1186/s12884-021-04199-1 (PMC8555341; doi:10.1186/s12884-021-04199-1)
Supplement: Supplementary file 1 — Additional file 1. English version questionnaire. [file 12884_2021_4199_MOESM1_ESM.pdf]

English version questionnaire

Incidence and determinants of adverse outcomes among women who were managed for Eclampsia in the University of Gondar Comprehensive Specialized Hospital, Northwest Ethiopia

This questionnaire was prepared for the collection of socio-demographic/clients' demography characteristics, clinical characteristics (clinical features, diagnostic/laboratory findings, maternal complications, clinical management/treatment, etc.), and outcome related information that are important for the assessment of incidence of AMOOs of Eclampsia and its determinant factors.

MRN ________________________ Date ________________________

No.	Questions with responses
101	Maternal age (document) …………………………
102	Marital status (select among the following)<br>A. Single<br>B. Married<br>C. Separated<br>D. Other, specify<br>E. Unknown status
103	Patient religion (select among the following)<br>A. Orthodox<br>B. Muslim<br>C. Protestant<br>D. Other, specify …………………
104	Place of residency (select and document)<br>A. Urban (Gondar, Keble……………………………<br>B. Rural (Woreda……………………Keble…………………
105	Maternal educational status (select among the following)

	A. Illiterate/no education<br>B. Primary education<br>C. Secondary education<br>D. Diploma/degree and above holders<br>E. Unknown
201	Maternal reproductive performance (document the finding)<br>A. Gravidity………………<br>B. Parity………………….<br>C. Abortion……………….<br>D. Not documented
202	Gestational age of current pregnancy (document) ……………………. in weeks<br>A. If not known, month of amenorrhea is ………………..…MOA<br>B. If Known the GA is ……………………………
203	Number of current pregnancy gestation (select among the following)<br>A. Singleton gestation<br>B. Multiple gestations.<br>C. If multiple gestations, write the numbers…………………..
204	Was there antenatal care visit?<br>A. No<br>B. Yes<br>C. If yes,<br>1. Numbers of ANC visits were...……………………...<br>2. Place of ANC visits were ……………………….
205	Were there danger/eminent symptoms prior to convulsion?<br>A. No, If no, go to Q. 207<br>B. Yes, If yes, go to the next Q.
	What were the danger symptoms/ eminent symptoms? (can be selected more than one)<br>1. Headache<br>2. Blurring of vision<br>3. RUQ pain<br>4. Epigastric pain

	5. Vomiting<br>6. History of raised blood pressure<br>7. Body swelling<br>8. Other, please specify …………………………………….
206	Document the duration of the above symptoms<br>………………………for ……………….duration<br>……………………...for ………………..duration
207	What was the convulsion duration before admission………………………………<br>What was the duration of convulsion in this hospital ………………………………..
208	What were the total numbers/episodes of convulsions ………………………………
209	Where were the places of convulsion? (can select more than one)<br>A. At home<br>B. At primary care<br>C. During transportation<br>D. At this hospital<br>E. Other, specify ………………………………..
210	When was the time of convulsion?<br>A. Antepartum<br>B. Intra-partum<br>C. Postpartum.<br>D. If PP, convulsion was after ………………………hrs./days of delivery
211	Was the convulsion uncontrolled?<br>A. No<br>B. Yes<br>C. If yes,<br>1. Was reloaded with MgSo4<br>2. Other anticonvulsants added, if yes, list the anticonvulsants added…………………………..<br>…………………………….………………………………………………………………….......................<br>3. Were convulsions during the follow and its duration after admission…………….…../ ……….....
212	The presence of failure to communicate in between or after convulsion<br>A. No, if no, go to Q. 214

	B. Yes, if yes, go to the next Q.
213	Duration of failure to communicate……………………………………………
214	Number health facilities visited for this complaint……………………………<br>A. Come directly or no referral<br>B. Arrived after one health facility visit<br>C. Arrived after two health facility visit<br>D. Other, specify
215A<br>215B	A. The total duration of stay in the primary care ………………………………………….<br>B. The total time taken from primary care to this hospital…………………………………
216	Anticonvulsants given before admission/at primary care<br>A. No<br>B. Yes, if yes, specify the medication……………………………………………………<br>What other treatment given there………………………………………………………..
217	Antihypertensive given before admission/at primary care<br>C. No<br>D. Yes, if yes, specify the medication……………………………………………………
218	Reason for referral…………………………………………………………………………..
301	Previous obstetric complications<br>A. No --- if no, go to No-302<br>B. Yes--- if yes, go the next Q.
	What were the previous obstetric complications? (can select more than one)<br>A. Abortion<br>B. Stillbirth<br>C. Preterm birth<br>D. Low birth weight<br>E. END<br>F. PIH<br>G. GDM<br>H. Other, specify …………………………………………………………………………..
302	Presence of coexisting personal-chronic illness<br>A. No, if no, go to Q. 303

	B. Yes, if yes, go to the next Q.
	What was the personal chronic Illness? (can select more than one)<br>A. Chronic hypertension<br>B. Diabetes mellitus<br>C. Chronic renal disease<br>D. Anemia<br>E. Other, specify …………………………………………………………………………………
303	The presence of known family-chronic illness<br>A. No<br>B. Yes<br>C. If yes, document the family illness ………………………………………………………
304	When was the previous last delivery, if there is any?<br>A. If known …………………………………...<br>B. Unknown/ not feasible
401	GCS at arrival/admission …………………………………………………….…………
402	A. Respiratory rate at admission ……………………………on follow-up ……………………<br>B. Oxygen saturation at admission ………………………..………………………………
403	A. Temperature at admission ……………………on follow up ………………………………<br>B. PR at admission ………………………on follow up ………………………………….........
404A<br>404B	A. Blood pressure at primary care……………………………………………………………<br>B. Blood pressure at admission to this hospital…………………………on follow up<br>…………………………. /………………..
405	Was there uncontrolled high blood pressure at primary care<br>A. No<br>B. Yes<br>C. If yes, answer Q. 701… on management part
406	Presence of body swelling at arrival at this hospital?<br>A. No<br>B. Yes,<br>C. if yes, (select among the following)<br>1. Is localized

	2. Is generalized<br>3. Has ascites
501	Laboratory results (document all results and tick the abnormal)<br>1. Urine Analysis<br>A. Proteinuria ………………………..<br>B. Ketone ……………………………<br>C. Glucose …………………………..<br>2. HCT…………………………………..<br>3. Platelet/Plt…………………………….<br>4. SGOT………………………………….<br>5. SGPT…………………………………..<br>6. Bil (D)…………………………Bil (T)………………….<br>7. PM……………………………………......<br>8. LDH……………………………………...<br>9. Creatinine…………………………….....<br>10. BUN………………………………………<br>11. Postpartum/ postop HCT……………………
502	Abnormal findings on Imaging? U/S, CT, MRI, CXR,…if A/B go to Q. 601<br>A. Not done<br>B. No abnormality<br>C. Yes, if yes, go to the next Q.
	Mark the abnormal findings<br>A. IUGR<br>B. Oligo-hydramnios<br>C. IUFD<br>D. Missed abortion<br>E. Others, specify…………………………………….
601	Labor initiation<br>1. Initiated spontaneously<br>A. Delivered spontaneously…………………………………………………..<br>B. Augmented with…. ……………………………………………………

	2. Induced, if induced answer the next Q. 602<br>3. Other, specify………………………………..
602	What were the induction methods used<br>A. Oxytocin<br>B. Misoprostol<br>C. Mifepristone<br>D. Mechanical dilators
603	What was the duration of labor?<br>A. Duration of labor before admission ……………………………in hours<br>B. Duration of labor after admission ……………………….………in hours
604	Mode of delivery<br>A. Vaginal delivery<br>B. Cesarean delivery
	1. Vertex vaginal delivery<br>2. Breech vaginal delivery<br>3. Instrumental vaginal delivery: Forceps, Vacuum, If yes, indication for instrumental was ………………………………………………….<br>4. Cesarean delivery<br>A. If yes, indication for CD is ……………………………………………………………<br>B. If CD, answer Q. 608 too<br>5. Hysterectomy if so why………………………………………………………………...
605	Place of delivery<br>A. Home delivery<br>B. Hospital delivery<br>C. Other, specify…………….
607	Intra-partum/ intraoperative findings ……………………………………………………………….
608	What was the type of cesarean delivery done? (If it is not CD, opt/ leave this Q.)<br>A. Primary CD before labor initiation<br>B. Intra partum CD during labor. If yes, for intra-partum CD, CD was done after …………hrs. of labor.
609	Write clinical features observed at admission ………………………………………………..

	During follow-up…………………………………………………………………………………
701	Was antihypertensive used for elevated blood pressure?<br>A. No<br>B. Yes,<br>C. If yes,<br>1. What antihypertensive used ……………………………………………………………………<br>2. Drug administration initiated after ……………hrs. of raised blood pressure. If late administration, why? ……………………………………………………………………...
702	Was anticonvulsant initiated/given?<br>A. No, if no, go to Q. 705<br>B. Yes<br>C. If yes, what is the drug?<br>1. MgSo4?<br>2. Other anticonvulsant given? Please specify the drug ……………………………………
703	Why other anticonvulsant drug used?<br>A. Added on MgSo4? If yes, after how long of MgSo4 treatment …… and Why? …..…………<br>B. Alternative to MgSo4? …………..if yes, why? ....<br>1. Due to toxicity of MgSo4<br>2. Due to lack of MgSo4<br>3. Other specify………………………………………………………………………
704	Was there MgSo4 toxicity?<br>A. No<br>B. Yes, if yes, answer the next Q.
	About MgSo4 toxicity<br>What was the toxicity……………………………………………………………………….<br>What was done? ……………………………………………………………………………<br>A. Dose reduced<br>B. Discontinued<br>C. Alternative anticonvulsant given<br>D. Calcium gluconate given<br>E. Other specify ………………………………………………………………………..

705	Antibiotics given?<br>A. No<br>B. Yes, if yes, list the antibiotics ……………………..<br>…………………………………………………………………………………………<br>And why given? …………………………………………………………………………...
706	Other treatment given? Specify ………………………………………………………………..
801	Blood transfusion requirement<br>1. No<br>2. Yes<br>3. If yes, what blood products …………………………………………………………………
802	Abruption placenta<br>A. No<br>B. Yes, if yes, specify the severity
803	Disseminated intravascular coagulation<br>A. No<br>B. Yes, if yes, document the evidence
804	Maternal shock?<br>A. No<br>B. Yes<br>C. If yes, what is the cause (mark from the following)<br>1. Retained placenta<br>2. Abruption<br>3. DIC<br>4. Sepsis<br>5. Other specify<br>D. If there was maternal shock, vasoactive agents used?<br>A. No<br>B. Yes,<br>C. If yes<br>1. Specify the vasoactive drug name ……………………………………………………….<br>2. Specify duration of use …………………………………………………………………..

	E. If there was maternal shock, was it corrected?<br>A. No<br>B. Yes.<br>C. If yes, with what agents?<br>1. Crystalloid fluids<br>2. Blood products<br>3. Vasoactive agents
805	Acute kidney injury?<br>A. No<br>B. Yes<br>C. Creatinine was not done<br>If yes, then answer the following Q. if not yes, go to Q. 806.
	1. Creatinine level<br>2. Oliguria<br>A. When<br>Antepartum?<br>Intrapartum?<br>Postpartum?<br>B. Duration of oliguria<br>C. Responded with fluid challenge/diuresis<br>No Yes<br>3. Has uremic manifestations<br>4. Dialysis done (A. No B. Yes)
806	HELLP (hemolysis elevated liver enzymes and low platelets syndrome)<br>A. No<br>B. Yes,<br>If yes, specify evidence with correct values……………………………………………….
807	Respiratory distress<br>1. No<br>2. Yes
808	Maternal cardiorespiratory condition

	A. Has heart failure<br>B. In cardiorespiratory distress<br>No, if no, go to Q. 809 Yes<br>C. If respiratory rate abnormal then duration of abnormality…………………………………<br>D. If oxygen saturation abnormal, specify for how long…..<br>E. Pulmonary edema (A. No B. Yes) …………………………………………………<br>F. CPR done A. No B. Yes<br>G. Aspiration pneumonia; if yes, for AP, then answer the next Q.
809	Evidence for aspiration pneumonia; at least (Specific chest findings? / High fever?) and another findings (mark among the following)<br>1. Has difficulty breathing/SOB<br>2. Has gasping<br>3. Has cyanosis<br>4. Has tachypnea<br>5. Has low O2 saturation<br>6. Other findings specify……………………………………………………………..
810	Maternal PPH<br>1. No<br>2. Yes<br>If yes,<br>A. Specify causes………………<br>B. Postpartum HCT………………….<br>C. Required transfusion?<br>A. No B. Yes, If yes, type of blood products transfused………………………………<br>Number of units transfused…………………
811	Neurologic complications<br>1. No<br>2. Yes<br>If yes,<br>A. Stroke<br>B. ICH

	C. Coma<br>D. Blindness<br>E. Other specify …………………………………………………………………………………
812	Intensive care unit admission/ High dependency area<br>1. No<br>2. Yes
813	Maternal death<br>1. No<br>2. Yes<br>If yes,<br>A. Cause of death ………..…………………………………………………………… B. When? 1. On arrival 2. ………. Hrs. /days of hospital stay
814	Specify, other maternal complications/adverse outcomes……………………………………………
901	Discharge status<br>A. Improved/recovered<br>B. Referred to………………………………………and why……………<br>C. Transferred<br>D. Disappeared/ went against/lost follow-up<br>E. Died<br>F. Other, specify
902	New admission 1. No 2. Yes
903	Admission date with time ____dd_____mm______ yyy, Time____________ PM/AM
904	Discharge date with time ____dd_____mm______yyy, Time____________ PM/AM
905	Hospital stay duration______________________________ in days
1001	Neonatal birth weight and sex …………………../…………………………………
1002	If it is abortion, put a mark here ……………. ………………
1003	Current stillbirth? (A. No B. Yes)<br>1. Freshly dead<br>2. Macerated, if yes, grading …………..<br>3. Cause of stillbirth?

	A. Abruption<br>B. Lethal congenital, Specify…………<br>C. Other specify ……………..
1004	Neonatal APGAR score (document the score if available)<br>A. Unknown but cried immediately……..<br>B. 1st min ……….<br>C. 5th min ………<br>D. 10th min……...if low APGAR, answer the next Q.
1005	Neonatal advanced life support/resuscitation given<br>A. No<br>B. Yes
1006	Neonatal status (mark among the following options)<br>A. Died in labor ward?<br>B. Admitted to NICU?<br>C. Stayed by mother side
1007	If neonate admitted to NICU, if not, go to Q. 908<br>1. Neonatal admission diagnosis……………………………………………………………<br>2. Gestational age by Ballard score assessment…………<br>3. Duration of stay at NICU<br>A. Known (specify) ... ……<br>B. Not known<br>4. Discharged<br>A. Improved<br>B. Against medical advice<br>C. Status not known<br>D. Status not feasible<br>E. END
1008	Cause of END (can mark more than one among the following)<br>1. RDS/HMD<br>2. IUGR<br>3. Abruption

	4. MAS<br>5. Sepsis<br>6. Prematurity<br>7. Other specify
1009	Perinatal conditions ………………………………………………………………………………<br>………………………………………………………………………………………………….
